# Prevalence of obesity and its relationship with lifestyle patterns among adults in Al-Baha, Saudi Arabia: A cross-sectional study

**DOI:** 10.1097/MD.0000000000047038

**Published:** 2026-01-16

**Authors:** Ramy H. Agwa, Turki H. Alkully, Twfiq A. Alghamdi, Rami F. Alghamdi, Ayman M. Alzahrani, Waleed S. Alghamdi, Abdulaziz M. Alomari, Adel A. Alghamdi, Abdulhakeem A. Alghamdi

**Affiliations:** a Department of Internal Medicine, Faculty of Medicine, Al-Baha University, Al-Baha, Saudi Arabia; b Hepatology and Gastroenterology Unit, Internal Medicine Department, Faculty of Medicine, Mansoura University, Mansoura, Egypt; c Department of Internal Medicine, Faculty of Medicine, Al-Baha University, Al-Baha, Saudi Arabia; d Al-Baha University, Al-Baha, Saudi Arabia.

**Keywords:** body mass index, lifestyle behaviors, obesity, public health, Saudi Arabia

## Abstract

Obesity, characterized by excessive body fat accumulation, is a significant global health problem and is increasingly prevalent in Saudi Arabia, largely due to lifestyle patterns and sociodemographic factors. This study aims to determine the prevalence of obesity and its association with lifestyle factors among adults in the Al-Baha region of Saudi Arabia. A cross-sectional study was conducted among 1008 adults aged ≥ 18 years in Al-Baha. Participants completed a validated, self-administered online questionnaire covering demographics, lifestyle behaviors, and health status. body mass index was calculated from self-reported height and weight. Lifestyle quality was scored and categorized. Data were analyzed using SPSS v26 with descriptive statistics and chi-square tests. Of the 1008 participants, 651 (64.6%) were males and 357 (35.4%) were females. Notably, obesity prevalence was higher among females (97, 27.2%) than males (92, 14.1%). The highest rate occurred in the 45 to 54-year-old age group (60, 35.7%). Obesity was observed in 119 (19.2%) university graduates and 10 (12.6%) postgraduates. Married participants (143, 22.7%) had a higher obesity rate than singles (40, 11.4%). A total of 114 (11.3%) were sedentary, and 54 (5.4%) consumed fast food daily. Most participants (740, 73.4%) had a fair lifestyle score, while only 11 (1.1%) reported excellent habits. Obesity was significantly associated with gender, age, marital status, education, occupation, and lifestyle score (*P* < .05). Obesity is highly prevalent in Al-Baha, particularly among women and middle-aged adults. The findings underscore the urgent need for targeted local programs promoting physical activity, healthier diets, and lifestyle education.

## 1. Introduction

Obesity is usually defined as abnormal or excessive fat accumulation that poses a health risk.^[1]^ Body mass index (BMI), defined as weight (kg) divided by square meter of height (m^2^), is an important recognized measure to evaluate this condition. Adults have widely applied BMI to classify their weight status using the following standard ranges: under 18.5 is considered underweight, while 18.5 to 24.9 is defined as normal, 25 to 29.9 as overweight, and ≥30 as obese.^[2]^

Obesity is a leading public health crisis because it affects virtually every physiologic system in the body.^[3]^ This greatly raises the odds of developing various chronic diseases, such as diabetes mellitus, cardiovascular disease, respiratory disease, many cancers, musculoskeletal disorders, and mental health problems.^[4]^ Obesity is a major global public health problem affecting more than 3 hundred million people worldwide. Its causes are complex and interrelated, involving genetic, epigenetic, physiological, behavioral, sociocultural, and environmental factors that eventually lead to a chronic imbalance between energy intake and energy expenditure.^[5]^ In 2022, according to the World Health Organization, 16% of adults (aged 18 years and older) globally were classified as obese. Notably, global adult obesity prevalence more than doubled from 1990 to 2022, and that of adolescents quadrupled.^[6]^

According to the Saudi Ministry of Health (2019), 20.2% of persons aged 18 and older were obese, while 38.2% were overweight. Women were slightly more likely than males to be obese (21.4%).^[7]^ This result shows the alarming necessity to combat obesity as a leading public health issue in the Kingdom. Factors contributing to lifestyles include physical inactivity (sedentary behaviors), food preferences and food intake, societal customs, gender roles, and environmental settings – all of which have worsened the obesity epidemic in Saudi society.^[8]^

Reviews of students at the Faculty of Applied Medical Sciences at Al-Baha University showed that 11% of obese people suffered from hypertension, 9% had diabetes, 7% had high cholesterol, and 14% had heart disease. Additionally, 20% of the obese students reported having sleep disorders.^[9]^

A study conducted to explore the prevalence of students’ obesity at the university found that 41.6% of the students were overweight or obese; namely, 23.8% were overweight, and 17.8% were obese. Specifically, the prevalence of overweight and obesity was highest among non-health-related students (37.7%), followed by health-related students (28.9%), while medical students had the lowest prevalence (22.6%). When health and non-health-related students were combined, the prevalence reached 66.6%. In addition, male students (34.5%) reported more than female students (24.9%).^[10]^

However, it is important to note that these studies were only conducted among university students. At the time this research was performed, no prior studies had been conducted to determine the prevalence of obesity and its association with lifestyle factors on a broader scale in the Al-Baha community. Consecutively, this study aimed to explore the prevalence of obesity and its relation to lifestyle patterns among adults in the Al-Baha region of Saudi Arabia.

## 2. Subjects and methods

A cross-sectional observational study was conducted to assess the prevalence of obesity and its association with lifestyle factors among adults in the Al-Baha region of Saudi Arabia. The study captured data at a single point in time, providing a descriptive overview of the current situation. A minimum sample size of 384 participants was required to achieve a 95% confidence level, as calculated using the Qualtrics sample-size calculator, to ensure statistical validity and precision.

Adults aged 18 years or older, of either sex, residing in Al-Baha or its governorates, and who provided informed consent were eligible for participation. Individuals younger than 18 years or not residing in the region were excluded. The questionnaire was disseminated through social media and community platforms across Al-Baha. Because participation was voluntary and the system allowed progress only after participants provided electronic consent, the total number of individuals who viewed the link but did not complete the survey could not be determined. Analyses were therefore limited to the 1008 fully completed responses. Recruitment covered both urban and rural areas to ensure regional representation. Self-reported survey responses may introduce recall bias and social desirability bias. Online recruitment may also contribute to selection bias.

Ethical approval for the study was obtained from the Scientific Research and Ethics Committee of Al-Baha University, Faculty of Medicine (IRB/MED/BU-FM/2024/110). The research was performed in compliance with the ethical principles outlined in the Declaration of Helsinki.

Data were collected using an author-designed, self-administered online questionnaire developed with the assistance of subject-matter experts to ensure content validity and reliability. A pilot test involving 53 adults was conducted to evaluate the questionnaire’s clarity, comprehensibility, and relevance. Feedback from the pilot was used to revise and refine the instrument by removing ambiguous and redundant items. The finalized questionnaire was distributed to participants, and data were collected between November and December 2024. It consisted of sections covering sociodemographic variables and sixteen items related to lifestyle behaviors, including dietary and fluid intake, physical activity, and social, mental, and overall health indicators. Completion of all items was mandatory to prevent missing data, and incomplete or ineligible responses were automatically excluded by the survey system. Missing data were not present because the online questionnaire required complete responses before submission.

Obesity was defined according to the World Health Organization classification as a BMI ≥ 30 kg/m^2^. Although newer diagnostic standards using imaging techniques such as dual-energy X-ray absorptiometry (DEXA) have been introduced, BMI remains the most widely used and internationally comparable indicator for obesity surveillance and population-based studies. Potential confounders considered included age, sex, marital status, education level, and occupation.

Data were entered into Microsoft Excel 2016 and analyzed using IBM SPSS Statistics version 26. Descriptive statistics were used to summarize participants’ characteristics and lifestyle behaviors. The sixteen lifestyle-related items were summed to produce an overall lifestyle-quality score for each participant. Scores were categorized as excellent (89–118 points), good (59–88), fair (30–58), or poor (0–29). Associations between categorical variables were assessed using chi-square tests, and statistical significance was set at *P* < .05. No multivariable regression analysis was performed; only chi-square tests were applied to assess associations. All reported associations are unadjusted bivariate findings.

## 3. Results

A total of 1008 adults from the Al-Baha region participated in the study. Table 1 presents their sociodemographic characteristics. Of these, 651 (64.6%) were males and 357 (35.4%) were females. Participants aged 25 to 34 years formed the largest group (288, 28.5%), followed by those aged 18 to 24 years (284, 28.2%). Most were university graduates (619, 61.4%), married (631, 62.6%), and living with family (916, 90.8%). Regarding residency, 343 (34.0%) lived in Al-Baha, while a smaller proportion were from Al-Hajrah (14, 1.4%). More than half (548, 54.4%) were employed full-time, and 361 (35.8%) reported a monthly income above 10,000 Saudi Riyal currency. Obesity was significantly associated with sex, age, education, marital status, and occupation (*P* < .05).

**Table 1:** Sociodemographic characteristics of participants.

Variables	Count	Percentage	χ^2^ (*P*-value)
Gender			
Male	651	64.6%	36.76 (*P* < .001)
Female	357	35.4%	
Age			
18–24	284	28.2%	97.14 (*P* < .001)
25–34	288	28.5%	
35–44	198	19.6%	
45–54	168	16.7%	
55–64	54	5.4%	
65 or older	16	1.6%	
Educational level			
Primary	9	0.9%	34.37 (*P* = .003)
Intermediate	13	1.3%	
High school	246	24.4%	
Diploma	41	4.1%	
University	619	61.4%	
Higher education	80	7.9%	
Residency			
Bani Hassan	40	4.0%	62.30 (*P* < .001)
Al Makhwah	121	12.0%	
Al Baha	343	34.0%	
Al Aqiq	64	6.3%	
Baljurashi	221	21.9%	
Qilwah	33	3.3%	
Al Hajrah	14	1.4%	
Al Qara	32	3.2%	
Al Mandaq	124	12.3%	
Ghamid Alzinad	16	1.6%	
Marital status			
Single	352	34.9%	59.64 (*P* < .001)
Married	631	62.6%	
Divorced	25	2.5%	
Living status			
Alone	56	5.6%	11.34 (*P* = .079)
With family	916	90.8%	
With friends	36	3.6%	
Occupation			
Student	209	20.7%	61.56 (*P* < .001)
Partial time employee	33	3.3%	
Full time employee	548	54.4%	
Unemployed	122	12.1%	
Retired	96	9.5%	
Monthly income			
<2000 SAR	244	24.2%	13.13 (*P* = .157)
2000–5000 SAR	90	8.9%	
5000–10,000 SAR	313	31.1%	
>10,000 SAR	361	35.8%	

SAR = Saudi riyal.

**Figure 1.**
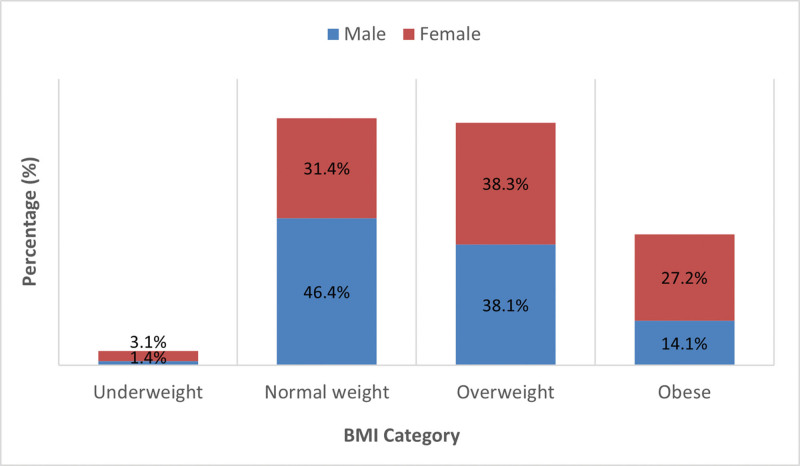
BMI distribution by sex. BMI = body mass index.

Figure 1 illustrates BMI distribution by sex. Among males, 302 (46.4%) had normal weight, 248 (38.1%) were overweight, 92 (14.1%) were obese, and 9 (1.4%) were underweight, while among females, 137 (38.3%) were overweight, 112 (31.4%) had normal weight, 97 (27.2%) were obese, and 11 (3.1%) were underweight. Overall, obesity and overweight were more frequent among females, whereas normal BMI was more common among males.

Table 2 summarizes participants’ lifestyle characteristics. A total of 440 (43.6%) reported no physical activity, and 44 (4.4%) exercised daily. Sedentary behavior was common: 352 (35.0%) reported sitting 4 to 6 hours per day and 337 (33.4%) 2 to 4 hours. Dietary patterns showed that 580 (57.5%) consumed 1 to 2 servings of fruits or vegetables daily and 534 (53.0%) ate fast food 1 to 2 times per week. For hydration, 711 (70.5%) drank 1 to 2 liters of water daily, while 152 (15.1%) consumed <1 liter. Regarding smoking status, 195 (19.3%) were current smokers, 51 (5.1%) were ex-smokers, and 762 (75.6%) had never smoked. Reported chronic conditions included diabetes (100, 9.9%), hypertension (75, 7.4%), and heart disease (17, 1.7%). Overall lifestyle quality was significantly associated with BMI categories (*P* < .001).

**Table 2:** Lifestyle habits of participants.

Variables	Count	Percentage	χ^2^ (*P*-value)
How many days per week do you engage in physical activity?			
0 d	440	43.6%	653.42 (*P* < .001)
1–2 d	334	33.1%	
3–4 d	165	16.4%	
5–6 d	25	2.5%	
Every day	44	4.4%	
How well you practice the following physical activities:			
Walking or running			
Never	285	28.3%	163.50 (*P* < .001)
Rarely	204	20.2%	
Sometimes	282	28.0%	
Often	172	17.1%	
Always	65	6.4%	
Cycling or motorcycle			
Never	725	71.9%	1747.37 (*P* < .001)
Rarely	130	12.9%	
Sometimes	107	10.6%	
Often	38	3.8%	
Always	8	0.8%	
Gym workouts			
Never	642	63.6%	1261.01 (*P* < .001)
Rarely	148	14.7%	
Sometimes	140	13.9%	
Often	58	5.8%	
Always	20	2.0%	
Playing sports (such as football and swimming)			
Never	705	69.9%	1617.87 (*P* < .001)
Rarely	134	13.3%	
Sometimes	111	11.0%	
Often	43	4.3%	
Always	15	1.5%	
On average, how many hours do you spend sitting (inactive) each day?			
>8 h	114	11.3%	351.14 (*P* < .001)
6–8 h	138	13.7%	
4–6 h	352	35.0%	
2–4 h	337	33.4%	
<2 h	67	6.6%	
How many servings of fruits and vegetables do you consume daily?			
0 servings	136	13.5%	656.99 (*P* < .001)
1–2 servings	580	57.5%	
3–4 servings	251	24.9%	
5 or more	41	4.1%	
How often do you consume fast food?			
Daily	54	5.4%	487.17 (*P* < .001)
3–4 times a week	194	19.2%	
1–2 times a week	534	53.0%	
Never	226	22.4%	
How many liters of water do you drink daily?			
<1 L	152	15.1%	627.86 (*P* < .001)
1–2 L	711	70.5%	
>2 L	145	14.4%	
Do you smoke?			
Yes	195	19.3%	841.02 (*P* < .001)
Ex-smokers	51	5.1%	
No	762	75.6%	
Do you consume any substances (such as alcohol or cocaine)?			
Yes	6	0.6%	1974.26 (*P* < .001)
Ex-consumer	1	0.1%	
No	1001	99.3%	
On average, how many hours of sleep do you get on a typical night?			
<4 h	28	2.8%	719.75 (*P* < .001)
4–6 h	540	53.6%	
6–8 h	372	36.9%	
>8 h	68	6.7%	
How often do you engage in social activities with friends or family (e.g., gatherings, outings)?			
Never	42	4.2%	359.94 (*P* < .001)
Rarely	374	37.1%	
Sometimes	414	41.0%	
Always	178	17.7%	
How would you rate your physical health in general?			
1	31	3.1%	727.94 (*P* < .001)
2	56	5.6%	
3	211	20.9%	
4	198	19.6%	
5	512	50.8%	
Have you ever been diagnosed with any of the following conditions? (select all that apply).			
Hypertension	75	7.4%	1817.87 (*P* < .001)
Diabetes	100	9.9%	
Heart diseases	17	1.7%	
None	859	85.2%	

Table 3 outlines motivations, barriers, and health-tracking behaviors. The most common motivator reported as “always” was improving physical appearance (141, 14.0%), followed by maintaining health (130, 12.9%) and enhancing physical fitness (124, 12.3%). The most commonly reported barriers were lack of time (71, 7.0%) and lack of motivation (57, 5.7%), followed closely by limited knowledge (53, 5.3%) and financial constraints (52, 5.2%). For health tracking, 62 (6.2%) participants always used mobile applications, and 61 (6.1%) used smartwatches or fitness trackers. Among perceived helpful resources, 174 (17.3%) strongly agreed on the usefulness of psychological and social support, 164 (16.3%) on exercise programs, and 153 (15.2%) on weight-control strategies. All variations were statistically significant (*P* < .001).

**Table 3:** Motivations, barriers, and resources for adopting a healthy lifestyle.

Variables	Count		Percentage	χ^2^ (*P*-value)
What is the following level of motivation to maintain or improve your weight?				
For health issues				
Never		88	8.7%	229.35 (*P* < .001)
Rarely		357	35.4%	
Sometimes		174	17.3%	
Often		259	25.7%	
Always		130	12.9%	
To improve body appearance				
Never		55	5.5%	350.71 (*P* < .001)
Rarely		179	17.8%	
Sometimes		413	40.9%	
Often		220	21.8%	
Always		141	14.0%	
To increase the level of physical fitness and activity				
Never		101	10.0%	136.34 (*P* < .001)
Rarely		283	28.1%	
Sometimes		254	25.2%	
Often		246	24.4%	
Always		124	12.3%	
Due to family or social pressures				
Never		362	35.9%	303.83 (*P* < .001)
Rarely		234	23.2%	
Sometimes		250	24.8%	
Often		120	11.9%	
Always		42	4.2%	
What level of obstacles do you face for not adopting a healthier lifestyle?				
Lack of time				
Never		116	11.5%	210.47 (*P* < .001)
Rarely		307	30.5%	
Sometimes		277	27.5%	
Often		237	23.5%	
Always		71	7.0%	
Lack of motivation				
Never		104	10.3%	340.26 (*P* < .001)
Rarely		260	25.8%	
Sometimes		388	38.5%	
Often		199	19.7%	
Always		57	5.7%	
Financial constrains				
Never		203	20.1%	212.85 (*P* < .001)
Rarely		313	31.0%	
Sometimes		281	27.9%	
Often		159	15.8%	
Always		52	5.2%	
Lack of awareness or knowledge				
Never		325	32.2%	243.65 (*P* < .001)
Rarely		233	23.1%	
Sometimes		272	27.0%	
Often		125	12.4%	
Always		53	5.3%	
What level of methods do you use to track your progress in managing your physical health?				
With some mobile apps				
Never		113	11.2%	261.48 (*P* < .001)
Rarely		285	28.3%	
Sometimes		337	33.4%	
Often		211	20.9%	
Always		62	6.2%	
Manual notes writing				
Never		216	21.4%	323.64 (*P* < .001)
Rarely		302	30.0%	
Sometimes		338	33.5%	
Often		125	12.4%	
Always		27	2.7%	
By fitness trackers or smart watches				
Never		211	20.9%	184.04 (*P* < .001)
Rarely		267	26.5%	
Sometimes		308	30.5%	
Often		161	16.0%	
Always		61	6.1%	
By conducting regular medical checkups				
Never		269	26.7%	258.02 (*P* < .001)
Rarely		305	30.2%	
Sometimes		272	27.0%	
Often		118	11.7%	
Always		44	4.4%	
What information do you think is most useful in adopting a healthier lifestyle?				
Nutrition education				
Strongly disagree		23	2.3%	723.94 (*P* < .001)
Disagree		67	6.6%	
Neutral		273	27.1%	
Agree		498	49.4%	
Strongly agree		147	14.6%	
Exercise programs				
Strongly disagree		18	1.8%	763.15 (*P* < .001)
Disagree		56	5.6%	
Neutral		262	26.0%	
Agree		508	50.3%	
Strongly agree		164	16.3%	
Weight control strategies				
Strongly disagree		25	2.5%	717.59 (*P* < .001)
Disagree		62	6.2%	
Neutral		272	27.0%	
Agree		496	49.1%	
Strongly agree		153	15.2%	
Psychological and social support importance				
Strongly disagree		29	2.9%	630.76 (*P* < .001)
Disagree		66	6.5%	
Neutral		265	26.3%	
Agree		474	47.0%	
Strongly agree		174	17.3%	
Stress management techniques (e.g., meditation, yoga)				
Strongly disagree		115	11.4%	381.27 (*P* < .001)
Disagree		111	11.0%	
Neutral		305	30.3%	
Agree		393	39.0%	
Strongly agree		84	8.3%	

**Figure 2.**
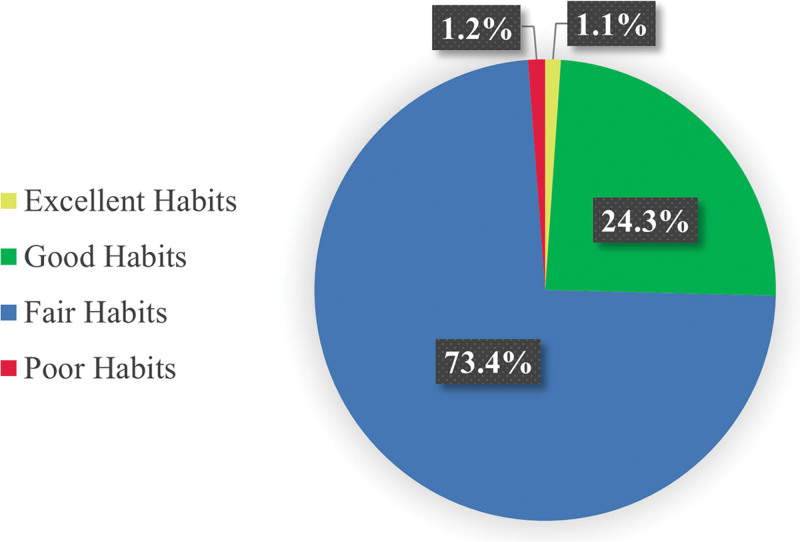
Distribution of participants across lifestyle quality categories.

Figure 2 displays the distribution of participants across lifestyle-quality categories. Most participants (740, 73.4%) had fair lifestyle habits, followed by 245 (24.3%) with good, 12 (1.2%) with poor, and 11 (1.1%) with excellent habits. These proportions describe the overall sample, while the BMI associations reported in Table 4 are based on the subset of participants with complete lifestyle-quality data.

**Table 4:** Association between obesity prevalence and demographic/lifestyle factors.

Variables	BMI								χ^2^ (*P*-value)
	Underweight		Normal weight		Overweight		Obese		
	Count	Percentage	Count	Percentage	Count	Percentage	Count	Percentage	
Gender									
Male	9	1.4%	302	46.4%	248	38.1%	92	14.1%	36.92 (*P* < .001)
Female	11	3.1%	112	31.4%	137	38.3%	97	27.2%	
Age									
18–24	15	5.3%	148	52.1%	92	32.4%	29	10.2%	97.11 (*P* < .001)
25–34	2	0.7%	126	43.7%	118	41.0%	42	14.6%	
35–44	2	1.0%	80	40.4%	78	39.4%	38	19.2%	
45–54	0	0.0%	38	22.6%	70	41.7%	60	35.7%	
55–64	0	0.0%	19	35.2%	20	37.0%	15	27.8%	
65 or older	1	6.2%	3	18.8%	7	43.8%	5	31.2%	
Educational level									
Primary	1	11.1%	0	0.0%	3	33.3%	5	55.6%	35.20 (*P* = .002)
Intermediate	0	0.0%	2	15.4%	4	30.8%	7	53.8%	
High school	5	2.0%	116	47.2%	88	35.8%	37	15.0%	
Diploma	0	0.0%	16	39.1%	14	34.1%	11	26.8%	
University	13	2.1%	247	39.9%	240	38.8%	119	19.2%	
Higher education	1	1.2%	33	41.2%	36	45.0%	10	12.6%	
Marital status									
Single	15	4.3%	181	51.3%	116	33.0%	40	11.4%	57.99 (*P* < .001)
Married	3	0.5%	229	36.3%	256	40.5%	143	22.7%	
Divorced	2	8.0%	4	16.0%	13	52.0%	6	24.0%	
Living status									
Alone	4	7.1%	23	41.1%	20	35.7%	9	16.1%	11.51 (*P* = .074)
With family	15	1.6%	376	41.0%	348	38.1%	177	19.3%	
With friends	1	2.8%	15	41.7%	17	47.2%	3	8.3%	
Occupation									
Student	13	6.2%	105	50.2%	71	34.0%	20	9.6%	61.36 (*P* < .001)
Partial time employee	1	3.0%	13	39.4%	15	45.5%	4	12.1%	
Full time employee	5	0.9%	218	39.8%	222	40.5%	103	18.8%	
Unemployed	0	0.0%	39	32.0%	41	33.6%	42	34.4%	
Retired	1	1.0%	39	40.6%	36	37.5%	20	20.9%	
Monthly income									
<2000 SAR	8	3.3%	114	46.7%	83	34.0%	39	16.0%	12.07 (*P* = .209)
2000–5000 SAR	3	3.3%	33	36.7%	32	35.6%	22	24.4%	
5000–10,000 SAR	4	1.3%	129	41.2%	122	39.0%	58	18.5%	
>10,000 SAR	5	1.4%	138	38.2%	148	41.0%	70	19.4%	
Lifestyle habits									
Poor	0	0.0%	5	41.7%	1	8.3%	6	50.0%	44.46 (*P* < .001)
Fair	20	2.7%	287	38.8%	270	36.5%	163	22.0%	
Good	0	0.0%	117	47.7%	108	44.1%	20	8.2%	
Excellent	0	0.0%	5	45.5%	6	54.5%	0	0.0%	

BMI = body mass index, SAR = Saudi riyal.

Table 4 shows the association between BMI categories and demographic and lifestyle variables. Obesity was more prevalent among females (97, 27.2%) than males (92, 14.1%) and peaked among those aged 45 to 54 years (60, 35.7%). University graduates represented the largest group among the obese (119, 19.2%). Married individuals (143, 22.7%) had higher obesity prevalence than singles (40, 11.4%). Obesity was more frequent among those living with family (177, 19.3%) and among full-time employees (103, 18.8%) than students (20, 9.6%) or unemployed participants (42, 34.4%). Monthly income was not significantly related to BMI (*P* = .209). Participants with fair lifestyle quality showed higher obesity rates (163, 22.0%) compared with those with good habits (20, 8.2%). Overall, BMI was significantly associated with sex, age, marital status, education, occupation, and lifestyle score (*P* < .05).

## 4. Discussion

This study aimed to determine the prevalence of obesity and its relationship with demographic and lifestyle factors among adults in the Al-Baha region. Among 1008 participants, the findings highlighted important behavioral and demographic patterns influencing obesity in this community. The observed prevalence aligns with earlier national studies, including a recent review reporting obesity rate in Saudi Arabia between 20% and 39%.^[11]^ Comparable prevalence rates have also been documented in Saudi Arabia and other countries,^[12–16]^ reinforcing the recognition of obesity as a global epidemic.

Significant differences were noted across demographic subgroups. Females demonstrated higher obesity prevalence than males (97, 27.2% vs 92, 14.1%), a trend consistent with prior Saudi studies.^[17–20]^ Cultural, environmental, and lifestyle factors, particularly those limiting women’s physical activity, may explain this disparity. Age also emerged as a strong predictor: obesity was most common among individuals aged 45 to 54 years (60, 35.7%), in line with previous studies linking prolonged sedentary behavior and reduced metabolic activity across different age groups.^[21–23]^

An inverse association was observed between education and obesity. Among participants with university degrees, 119 (19.2%) were obese, compared to 10 (12.6%) among those with postgraduate degrees. This finding is consistent with previous studies showing that higher educational attainment correlates with better health literacy, healthier dietary habits, and greater physical activity.^[24,25]^ Marital status was also relevant, with 143 married participants (22.7%) classified as obese compared to 40 singles (11.4%). Lifestyle changes after marriage, including reduced physical activity and increased caloric intake, may account for this difference.^[26]^

Lifestyle quality showed a strong association with obesity. Those in the “fair” lifestyle category had 163 obese participants (22.0%), compared to 20 (8.2%) in the “good” lifestyle category, supporting prior research on modifiable behaviors and weight regulation.^[27–29]^ Low physical activity was common, with 440 participants (43.6%) reporting inactivity, while 534 (53.0%) consumed fast food 1 to 2 times per week. These behaviors are known contributors to excessive energy intake and weight gain.^[30,31]^ Smoking prevalence was relatively low (195 participants, 19.3%), but its relationship with body weight remains complex. While smoking may suppress appetite in some individuals, its overall health risks far outweigh any potential weight-related effects.^[32]^

Psychosocial motivators and barriers also shaped lifestyle behaviors. A total of 141 participants (14.0%) reported improving physical appearance as a key motivator, while 71 (7.0%) consistently cited lack of time as a barrier. Lack of motivation (57, 5.7%), lack of knowledge (53, 5.3%), and financial challenges (52, 5.2%) were also reported. These findings align with international literature highlighting similar behavioral challenges.^[33,34]^ Such results suggest that individual interventions must be supported by broader systemic strategies to address persistent barriers.

Similar findings have been reported in other Gulf countries such as Kuwait, Bahrain, and the United Arab Emirates.^[35,36]^ At the global level, obesity prevalence has risen dramatically over recent decades across both developed and developing nations, driven by increasingly sedentary lifestyles and high-calorie diets.^[37]^ In Saudi Arabia, the government’s Vision 2030 Health Sector Transformation Program emphasizes preventive health strategies, lifestyle modification, and community engagement to reduce the national burden of obesity.^[38]^ These findings highlight the relevance of the present study to both regional and national health priorities.

This study has limitations. The cross-sectional design prevents causal inference, and reliance on self-reported data may introduce recall or reporting bias. In addition, the use of an online self-reported questionnaire may have introduced recall and selection bias, potentially favoring more health-conscious individuals. Moreover, the analysis relied solely on bivariate comparisons without multivariable regression modeling; as a result, confounding factors such as age, sex, or socioeconomic status may have influenced the observed associations. Additionally, obesity prevalence was not stratified across every exposure category, limiting the depth of association testing. Future research should incorporate longitudinal designs and more detailed subgroup analyses to strengthen causal inference. Finally, while the findings provide valuable insight into obesity in Al-Baha, they may not be fully generalizable to other Saudi regions due to cultural and lifestyle differences.

Overall, this study contributes to the growing body of evidence on obesity epidemiology in Saudi Arabia and provides locally relevant data for the Al-Baha region. The results underscore the urgent need for targeted interventions focusing on lifestyle education, social access to physical activity, and at-risk groups. Aligning such initiatives with the Vision 2030 preventive-health objectives could substantially improve population health outcomes across the Kingdom.

## 4.1. Conclusion

This study involved 1008 participants and found a high prevalence of obesity in the Al-Baha region, particularly among female, middle-aged, and less educated individuals. Physical inactivity was positively associated with an increased incidence of obesity, as well as an increased frequency of fast-food consumption and prolonged inactivity. Although they had health-related reasons for doing so, challenges, such as lack of time and motivation, were preventing them from putting healthy behaviors into practice. The results underscore the urgent need for targeted interventions focusing on lifestyle education, social access to physical activity, and at-risk groups. Further studies should be conducted to assess the long-term outcomes and efficacy of any interventions.

## Author contributions

**Conceptualization:** Twfiq A. Alghamdi, Rami F. Alghamdi.

**Data curation:** Twfiq A. Alghamdi, Waleed S. Alghamdi.

**Formal analysis:** Twfiq A. Alghamdi, Ayman M. Alzahrani.

**Investigation:** Twfiq A. Alghamdi, Rami F. Alghamdi, Ayman M. Alzahrani, Waleed S. Alghamdi, Abdulaziz M. Alomari, Adel A. Alghamdi, Abdulhakeem A. Alghamdi.

**Methodology:** Abdulaziz M. Alomari.

**Project administration:** Ramy H. Agwa, Turki H. Alkully.

**Resources:** Adel A. Alghamdi, Abdulhakeem A. Alghamdi.

**Supervision:** Ramy H. Agwa, Turki H. Alkully.

**Validation:** Ramy H. Agwa, Turki H. Alkully.

**Writing – original draft:** Twfiq A. Alghamdi, Rami F. Alghamdi, Ayman M. Alzahrani, Waleed S. Alghamdi, Abdulaziz M. Alomari, Adel A. Alghamdi, Abdulhakeem A. Alghamdi.

**Writing – review & editing:** Twfiq A. Alghamdi.
